# Complete genome of the *Alkalibacterium* sp. f15 isolated from Daihai Lake in Inner Mongolia

**DOI:** 10.1128/mra.00356-25

**Published:** 2025-08-27

**Authors:** Kai Jiang, Gen Che, Bo Yuan, Chunling Cao, Yanxing Wang, Lijia Ye, Yu Hong

**Affiliations:** 1 College of Life Science and Technology, Inner Mongolia Normal University 71203 , Hohhot, Inner Mongolia, China; 2 Key Laboratory of Biodiversity Conservation and Sustainable Utilization in Mongolian Plateau for College and University of Inner Mongolia Autonomous Region, Hohhot, China; 3 Department of Agriculture and Animal Husbandry of Inner Mongolia, Hohhot, Inner Mongolia, China; University of Guelph, Guelph, Canada

**Keywords:** *Alkalibacterium*, Daihai Lake, complete genome, 16S rRNA gene

## Abstract

We isolated a strain of the genus *Alkalibacterium* from Daihai Lake in Inner Mongolia. The complete genome size is 2,614,554 bp, with a genomic DNA G + C content of 39.3%. Metabolic pathway analysis indicates that strain f15 is a potential lactic acid-fermenting bacterium with industrial applications in lactate fermentation.

## ANNOUNCEMENT

The genus *Alkalibacterium* was first described by Spyridon Ntougias and Nicholas J. Russell in 2001, with its type strain, *A. olivapovliticus* sp. WW2-SN4a^T^ isolated from the wash water of edible olives (1). Currently, 10 species from diverse habitats like water, sediment, and soil (1–7) have been published. *Alkalibacterium* species are rod-shaped, occurring singly, in pairs, in clusters, or in short chains. They are non-sporulating and facultatively anaerobic. Oxidase and catalase reactions are negative (1). Most strains are motile by peritrichous flagella, but a few of the strains, such as *A. gilvum* (3), *A. thalassium* (4), *A. subtropicum* (6), are non-motile. All species within the genus *Alkalibacterium* possess halophilic and alkaliphilic characteristics and produce lactic acid as the primary product of glucose fermentation (6).

Strain f15 was isolated from the surface water of Daihai Lake (pH 9.0, 17°C, salinity 1.2%). The sample was serially diluted with sterile lake water, and the 10^−1^ dilution was filtered through 0.22 µm membrane for inoculation. Dilutions (100 µL) were spread onto six different solid media and incubated at room temperature for over one month. Pure f15 colonies were obtained by streak purification based on colony morphology and phenotypic characteristics.

The f15 strain was cultured in Luria-Bertani agar broth to logarithmic phase, and genomic DNA was extracted with the Blood & Cell Culture DNA Kit (QIAGEN, Germany). Size-selected DNA (BluePippin) was end-repaired, barcoded (NBD103/114), adapter-ligated, and quantified (Qubit) for library preparation. Sequencing was conducted by Nextomics Biosciences (Wuhan, China) using both Oxford Nanopore Technology (8) (PromethION platform) and Illumina NovaSeq 6000 platform (9). FAST5 files were basecalled to FASTQ using Guppy v3.1.5 (10), filtered for adapters/errors, and quality-selected (Q ≥ 7) to obtain assembly-ready pass reads. The raw data were assembled with Flye v2.7 (--plasmids --nano-raw) (11, 12), and corrections were performed with Pilon v1.23 (13) combined with second-generation sequencing data (Next-Generation Sequencing [NGS]), and Racon v1.4.13 (14) combined with third-generation sequencing data (Oxford Nanopore Technologies [ONT]). The corrected genome was circularized using custom scripts, trimmed of redundant regions, and standardized with Circlator v1.5.1 (--fixstart) (15) to reorient the origin at the replication start site. Genome annotation was conducted using the NCBI Prokaryotic Genome Annotation Pipeline (16). The complete 16S rRNA gene sequence was obtained from whole genome data using RNAmmer v1.2 (-S bac) (17). The phylogenetic tree was constructed using MEGA v7.0 (18), applying the maximum-likelihood (ML) method (19). Default parameters were used except where otherwise noted.

**Fig 1:**
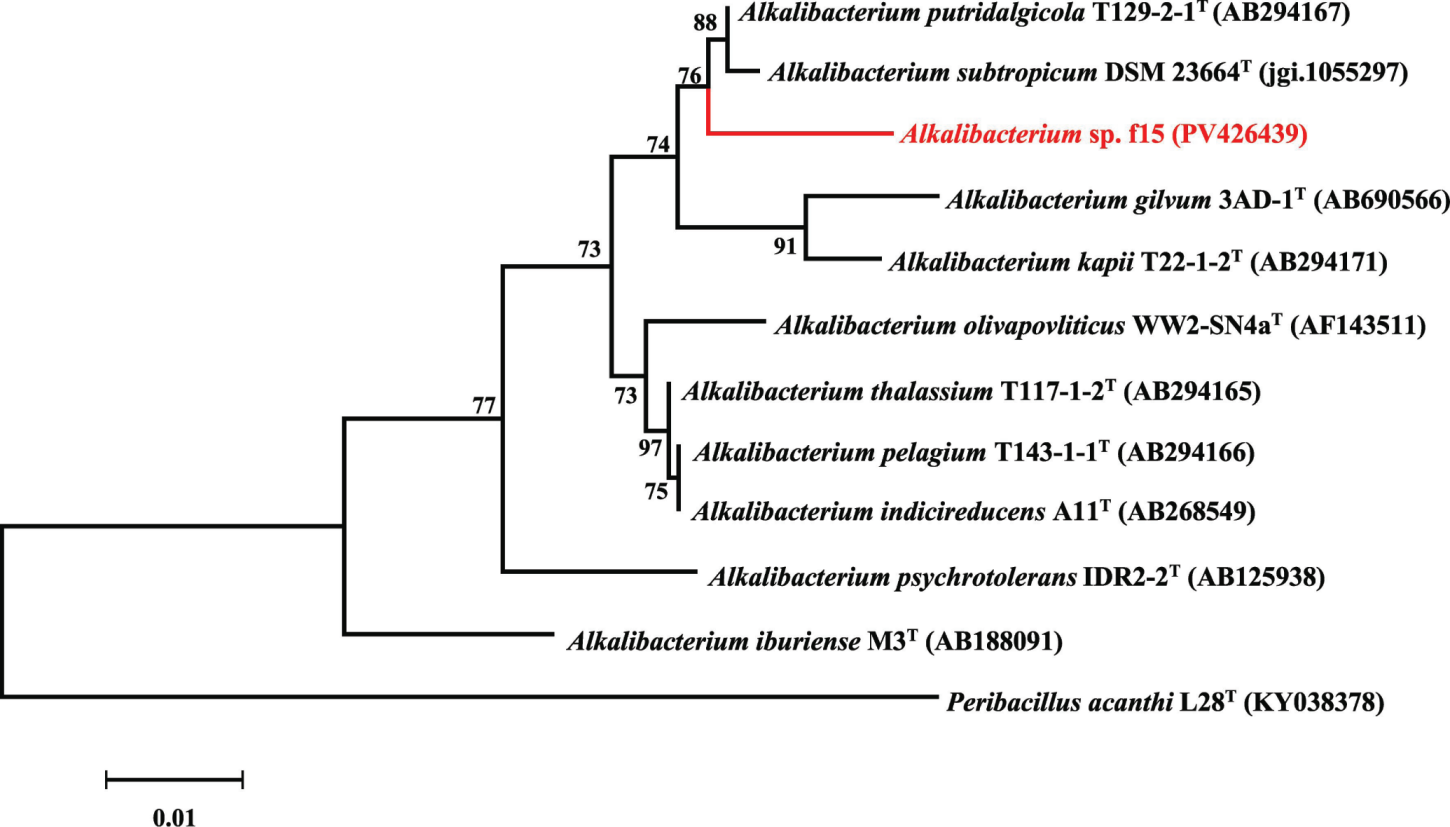
Maximum-Likelihood tree based on 16S rRNA gene sequences using the Kimura 2-parameter method, showing the phylogenetic position of strain f15 within related genera. Bootstrap values were based on 1,000 replicates (values ≥ 50% were shown). *Peribacillus acanthi* L28^T^ was used as the out-group. Bar, 0.01 changes per nucleotide position.

In this study, strain f15 generated a total of 1.176 Gb of raw data after sequencing, which was reassembled into a final complete contig of 2,614,554 bp, with a coverage depth exceeding 415-fold. The genomic DNA G + C content was 39.3%. The genomic data, assembly, and annotation information of strain f15 are summarized in Table 1. The length of the 16S rRNA gene is 1,542 bp, and the 16S rRNA phylogenetic tree indicates that strain f15 belongs to the genus *Alkalibacterium* (Fig. 1). It shows the highest similarity of 98.4% with the type strain *A. putridalgicola* T129-2-1^T^, which is lower than the threshold of 98.7%.

**TABLE 1:** The genomic data, assembly, and annotation information of strain f15^*a*^

Project	ONT	NGS
Raw reads	1,176,841,583 bp	1.25 Gb
Filtered reads	1,131,024,195 bp	1.24 Gb
Reads N50	24,946 bp	–^*b*^
Longest reads	138,508 bp	–
Genome size	2,614,554 bp	–
Q20	–	98.26%
Q30	–	84.37%
Genomic DNA G + C content	39.31%	39.54%
Contig number	1	
Sequencing depth (X)	415.47	
CDS	2,353	
tRNA	74	
rRNA	19	

^*a*^ ONT (Oxford Nanopore Technologies): third-generation sequencing data; NGS (Next-Generation Sequencing): second-generation sequencing data.

^*b*^ –, no data available.

## Data Availability

The whole-genome data of Alkalibacterium sp. f15 can be obtained under the accession CP186540 in GenBank. The 16S rRNA gene sequence has been deposited in GenBank, and the accession number is PV426439. The Sequence Read Archive (SRA) accession numbers for raw reads are SRR33065459 (NGS) and SRR33065460 (ONT), respectively. The BioSample and BioProject accession numbers are SAMN47742806 and PRJNA648510, respectively.
